# Bilateral congenital muscular torticollis in infants, report of two cases

**DOI:** 10.12688/f1000research.143499.1

**Published:** 2024-03-21

**Authors:** Anna Öhman

**Affiliations:** 1 Health and Rehabilitation/Physiotherapy, University of Gothenburg, Gothenburg, Sweden

**Keywords:** Torticollis, bilateral, infant, physiotherapy

## Abstract

**Background:**

Congenital muscular torticollis (CMT) is a well-known diagnosis among physiotherapists specializing in pediatric care, especially when working with infants. However, knowledge of bilateral torticollis is limited. The purpose of this article was to describe how bilateral torticollis can appear in infants.

**Case:**

One infant with CMT with sternocleidomastoid tumor (SMT) on the right side, and some limitation in rotation towards the right side and in lateral flexion towards the left side, that is, the muscle on the right side was shortened. While sitting with support, he tilted the head to the left and was stronger in the lateral flexors on the left side which fit well with a postural left-sided torticollis (PT). The other infant had bilateral muscular torticollis (MT), the sternocleidomastoid muscle thickened bilaterally, and both active and passive rotations were affected. The head was held in flexion, and active rotation was severely limited to both sides. For both cases the therapeutic interventions were to gain a normal range of motion (ROM) and a good posture of the head.

**Conclusions:**

CMT can appear in different ways and may be bilateral. Both infants gained good ROM and better head position, however case I still needs some training. To gain more knowledge about bilateral CMT, we should follow these cases over a longer period of time. It is important to communicate and discuss our experiences with each other to understand rare cases of CMT.

## Introduction

Congenital muscular torticollis (CMT) is a common congenital musculoskeletal anomaly among infants. CMT results from shortening or excessive contraction of the sternocleidomastoid (SCM) muscle. A sternocleidomastoid tumor (SMT) may be visible one to four weeks after birth, which consists of fibrous tissue and disappears within a few months ( Cheng, Tang and Chen, 1999; Kurtycz, Logrono, Hoerl and Heatley, 2000; Tang *et al.*, 2002; Wei, Schwartz, Weaver and Orvidas, 2001; Ohman and Beckung, 2008). The head is typically tilted towards the affected muscle, and the chin is rotated towards the other side. The true etiology of CMT have been discussed; one hypothesis is that the condition could be the sequel of an intrauterine or perinatal compartment syndrome ( Davis, Wenger, Mubarak, 1993), several studies are pointing in that direction ( Bielski *et al.*, 2006; Hardgrib, Rahbek, Möller-Madse and Maimburg, 2017; Lee *et al.*, 2011a, 2011b; Pazonyi, Kun and Czeizel, 1982; Xiong *et al*., 2019). CMT can be divided into three groups: I SMT group with a clinically palpable sternomastoid tumor; II muscular torticollis group (MT) with tightness of the SCM muscle; and III postural torticollis group (PT) with all the clinical features of torticollis, but without the tightness or tumor of the SCM muscle ( Cheng *et al.*, 2001). Treatment for CMT is aimed to prevent facial and skull asymmetry, neck movement limitation, and long-term posture change. Treatment includes active positioning, active range of motion (ROM), stretching of the shortened muscle, strengthening of the contralateral muscle, and surgery.

Bilateral torticollis is rare and not often reported, Matuszewski, Pietrzyk, Kandzierski and Wilczynsk (2017) reported a case of a child with bilateral torticollis, referred to the orthopedic department at the age of 12 years. The first symptoms appeared at preschool age. This child had severe limitations in range of motion and required bilateral surgery ( Matuszewski, Pietrzyk, Kandzierski and Wilczynsk, 2017). Babu *et al.*, presented a case report of a 19-year-old girl with congenital bilateral sternocleidomastoid contracture ( Babu, Lee, Mahadev and Lee, 2009).

A few articles describing case reports of bilateral torticollis have not been published in English ( Chiari, 1952; Kustos and Magdics, 1993; Shi *et al*., 2016; Shinoda and Yamada, 1969).

## Case I

**Figure 1.**
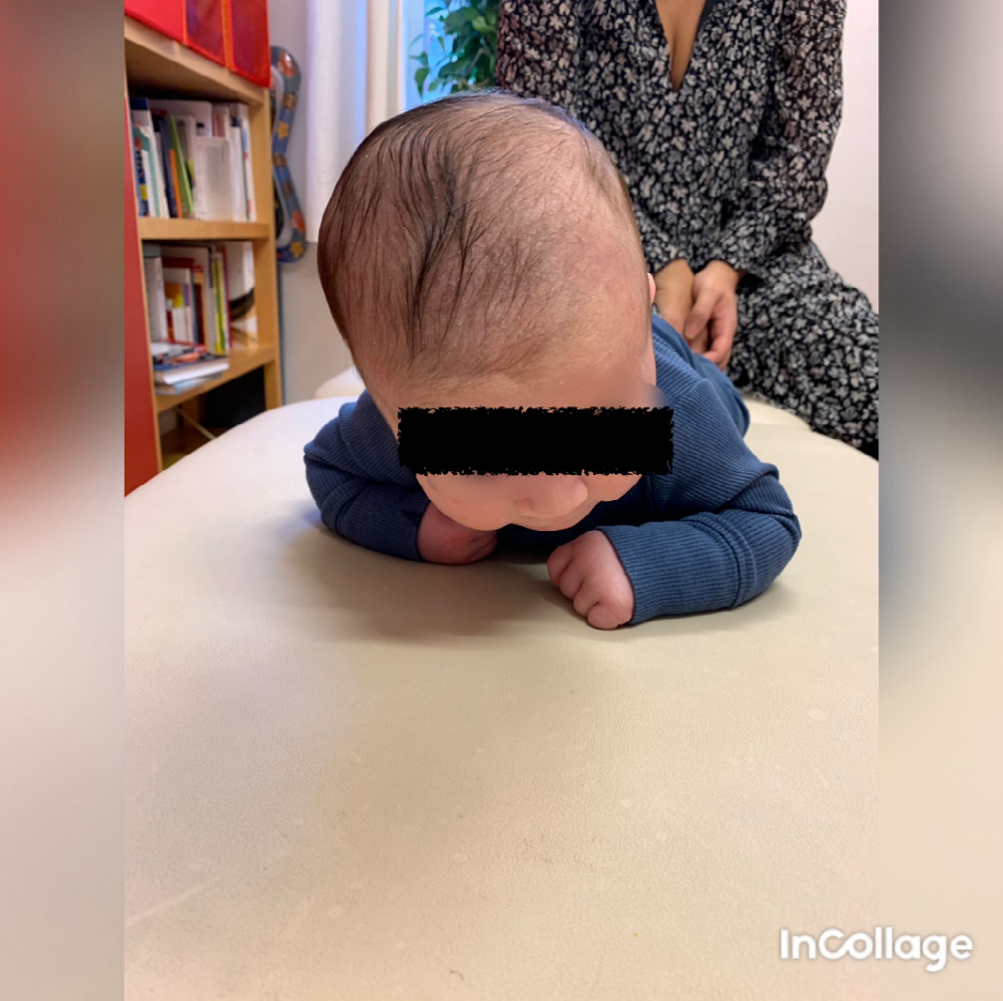
Case I, in the prone position, showed lateral flexion of the head to the right and rotation to the left. This is in agreement with right-sided torticollis. I confirm that I have obtained permission to use this image from the parents of the patient included in this presentation.

**Figure 2.**
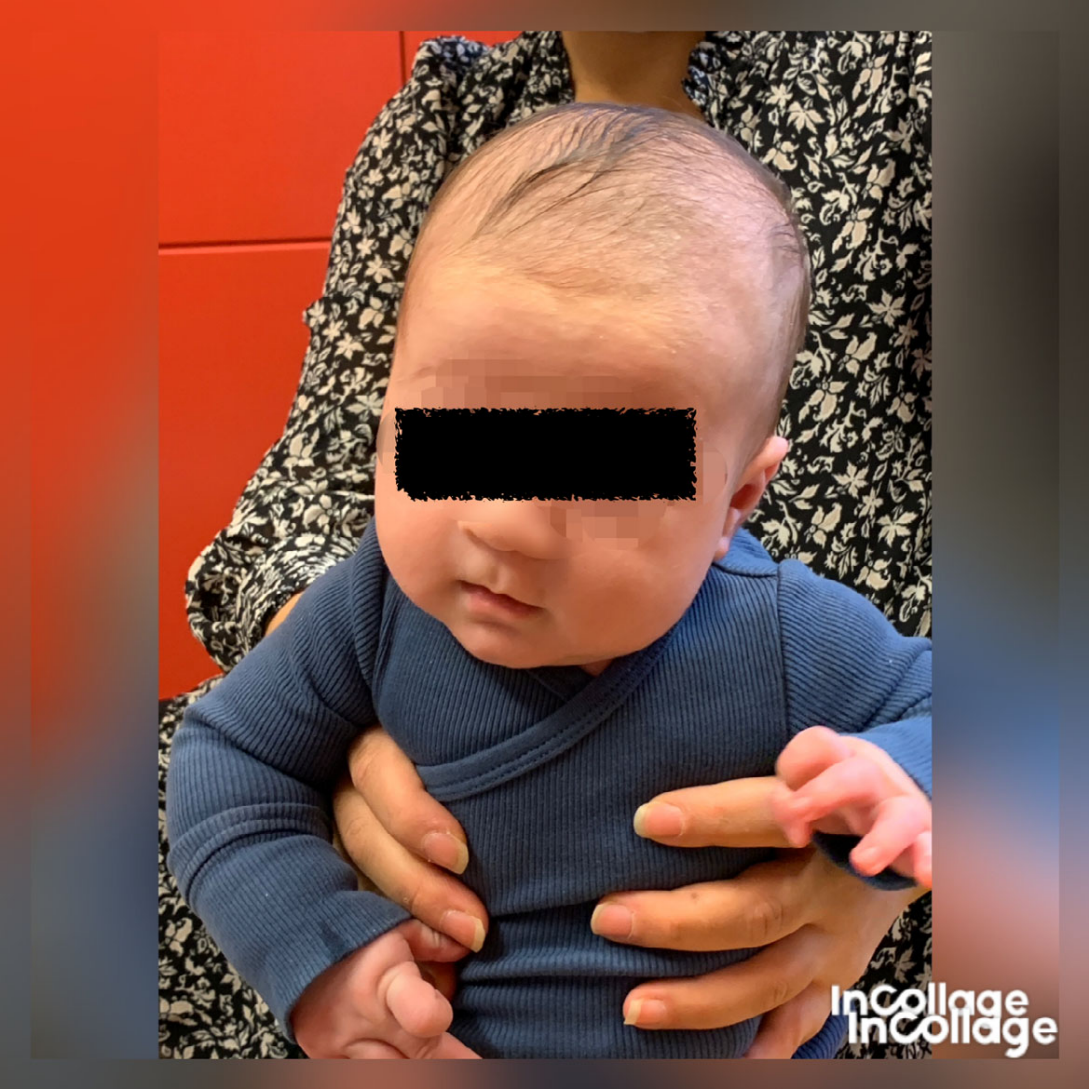
Case I in supported sitting position, lateral flexion of the head to the left and rotation to the right. This is agreeable with left-sided torticollis. I confirm that I have obtained permission to use this image from the parents of the patient included in this presentation.

**Figure 3.**
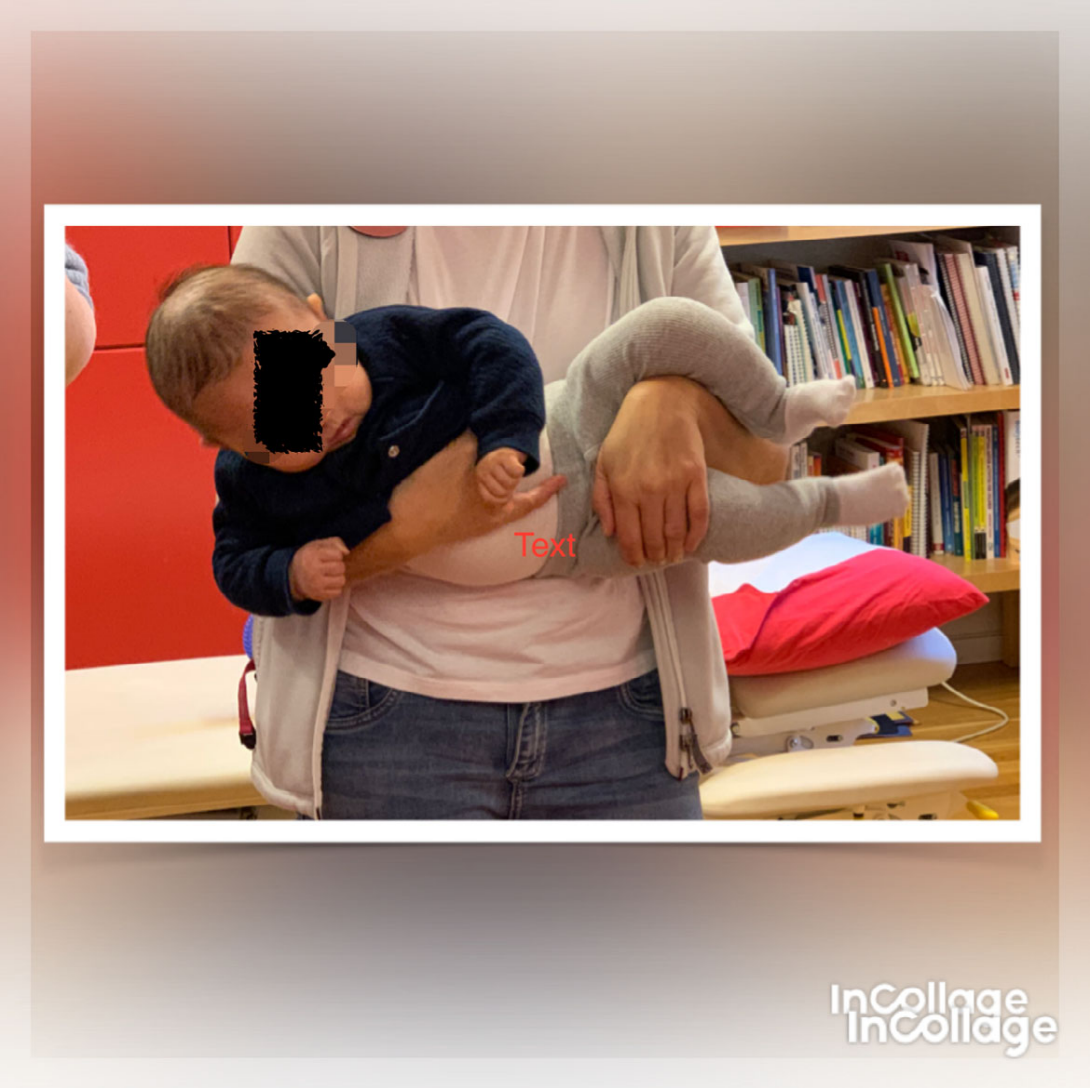
Case I test with the Muscle Function Scale (MFS) left side scoring 3, *i.e.,* left side is stronger than the right side. This is agreeable with left-sided torticollis. I confirm that I have obtained permission to use this image from the parents of the patient included in this presentation.

**Figure 4.**
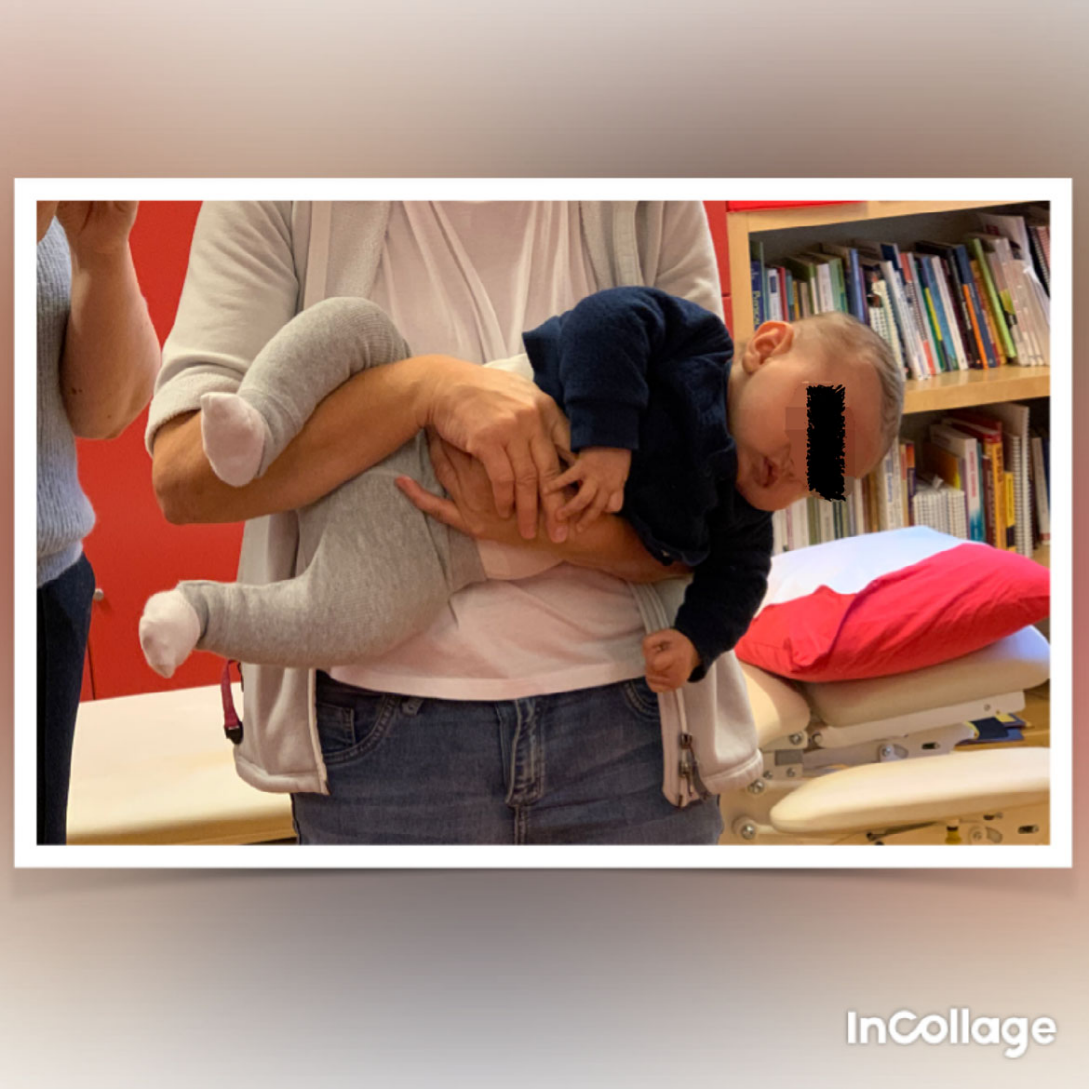
Case I test with the Muscle Function Scale (MFS) right side scoring 2, *i.e.*, right side is weaker than the left side. This is agreeable with left-sided torticollis. I confirm that I have obtained permission to use this image from the parents of the patient included in this presentation.

**Figure 5.**
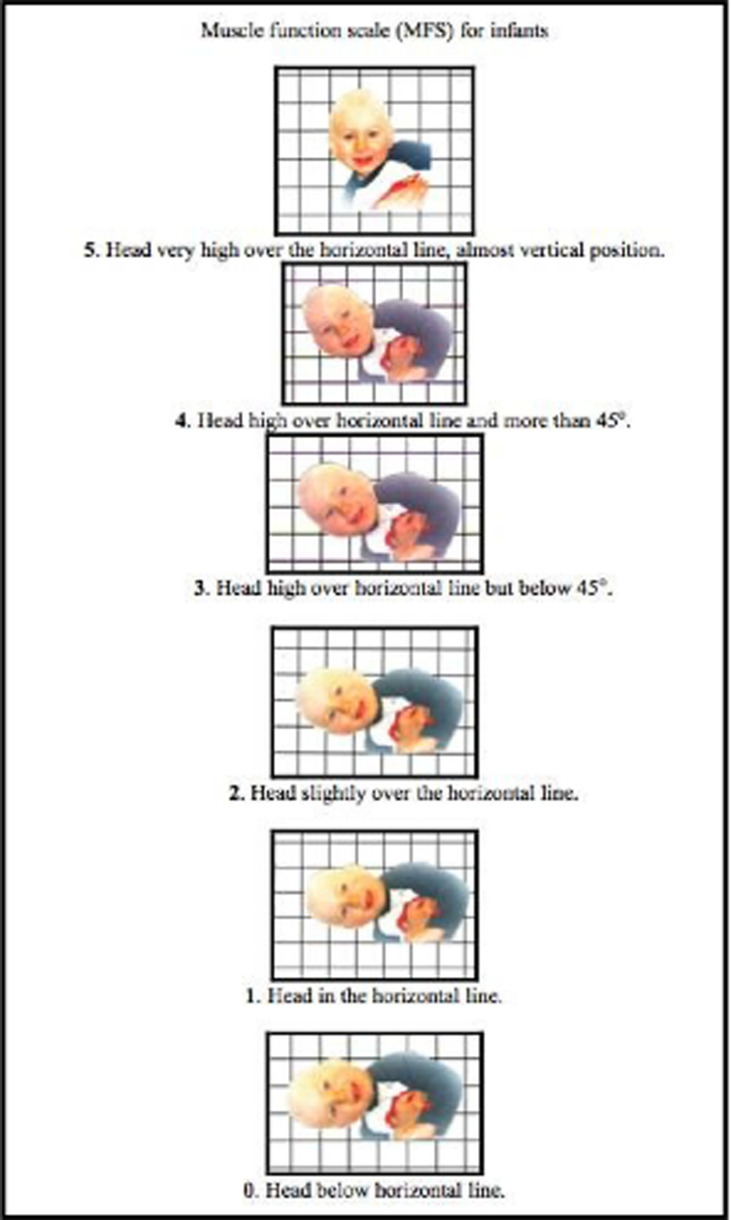
Muscle Function Scale. When the Muscle Function Scale (MFS) is used, the infant is held in a vertical position and then lowered to the horizontal position in front of a mirror. The head position is observed and both sides are tested. Scores are given according to the head position in relation to the horizontal line. The infant must be observed with the head held in the same position for five seconds to obtain the score at that level. This figure has been reproduced/adapted from Ohman *et al.* (2009). Reprinted by permission of Informa UK Limited, trading as Taylor & Francis Group, www.tandfonline.com.

Case I was a Caucasian boy (3.5 months of age) who came to Friskispraktiken, a physiotherapy clinic, in Gothenburg, Sweden for a second opinion since the first examiner found it confusing, when the motion, muscle function/strength, and tilting of the head did not clearly fit left- or right-sided torticollis. I met this boy in October 2020. When he was 3.5 months of age, he had bilateral torticollis. On the right side, the patient had a sternocleidomastoid tumor and some limitation in rotation towards the right side and in lateral flexion towards the left side, that is, the muscle on the right side was shortened. When lying in the supine or prone position, he tilted the head to the right side and rotated to the left ( Figure 1), which fit well with right-sided torticollis, SMT. While sitting with support, he tilted the head to the left ( Figure 2) and was stronger in the lateral flexors on the left side ( Figures 3 and 4), which fit well with a postural left-sided torticollis (PT). Muscle function and strength were measured using the Muscle Function Scale (MFS) ( Figure 5) ( Ohman and Beckung, 2008; Ohman, Nilsson and Beckung, 2009).

Treatment involved stretching of the muscle on the right side, strength training on the right side as the left side was stronger, and tilting the head to the left in a vertical position. The treatment at that time gave quick results, equal strength in the lateral flexors of the neck, motion in lateral flexion, and rotation only a marginal difference.

At the follow-up at two years of age, he had relatively good motion and no head tilt, but he had an indication of a discreet muscular string on the right side. At the next follow-up at two and a half years of age, he had a discreet tendency to tilt the head to the right and was slightly stronger on the right side. The muscular string is still rather discreet and felt only when stretching the muscle. He has started treatment again, and the muscular string may worsen as he grows in height. The SCM muscle grows from about 4 cm in infants to 14 cm at 13 years of age, according to measures made by Jones (1968). Case I must be followed-up for a longer time, as there is a risk that he will need surgery later in life.

## Case II

**Figure 6.**
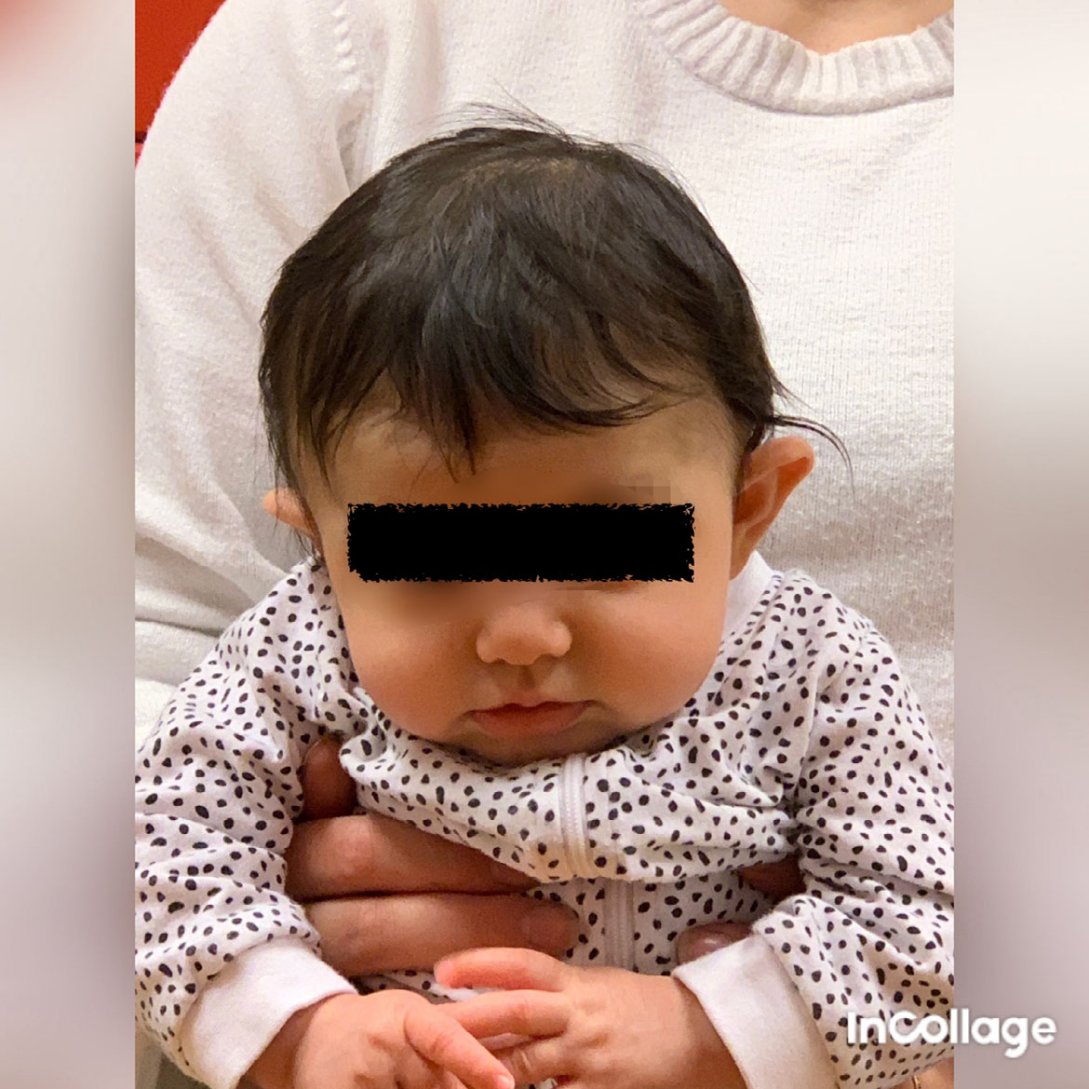
Front view of Case II, head in flexion due to bilateral torticollis. I confirm that I have obtained permission to use this image from the parents of the patient included in this presentation.

**Figure 7.**
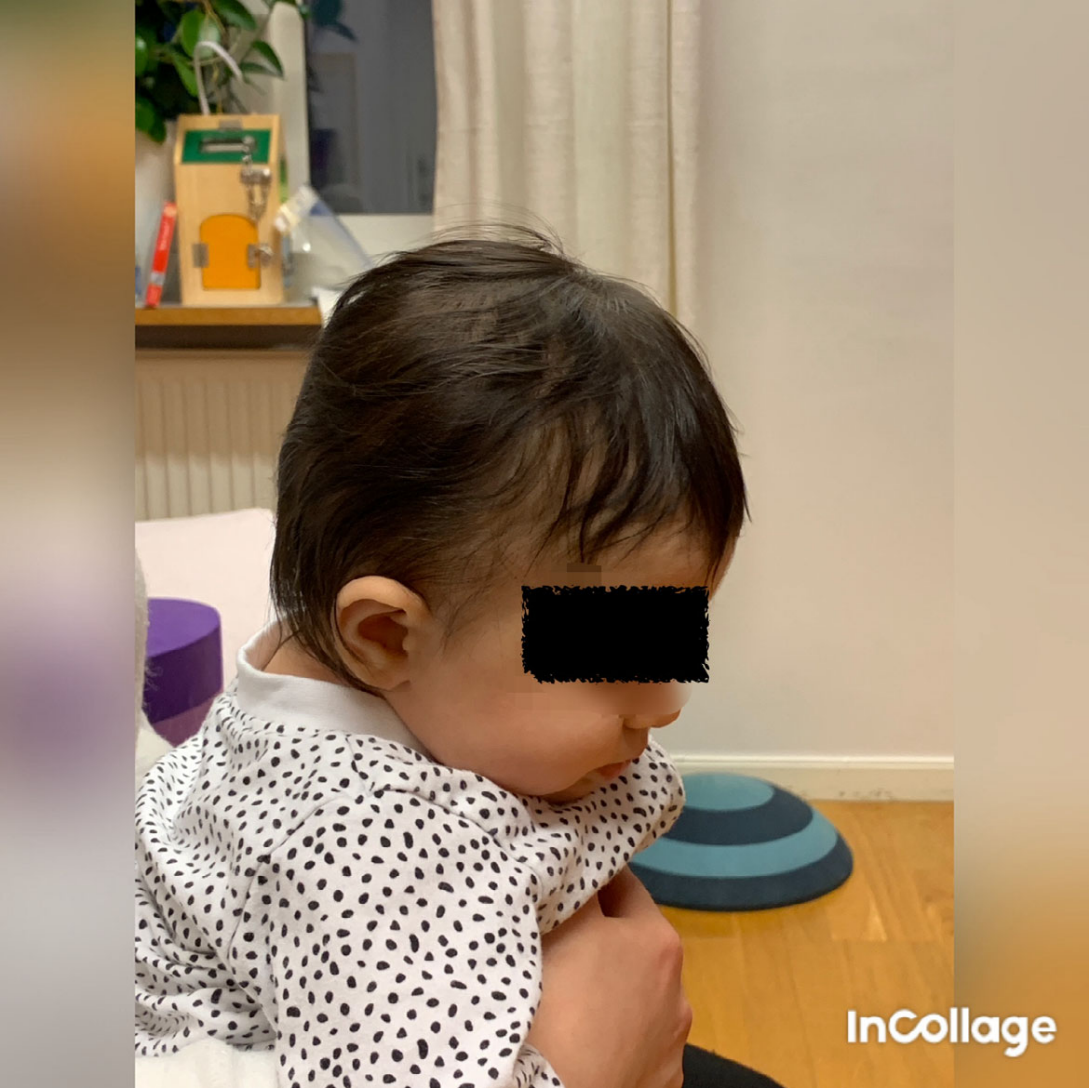
Side view of Case II, head in flexion and only attempts to rotate the head. I confirm that I have obtained permission to use this image from the parents of the patient included in this presentation.

**Figure 8.**
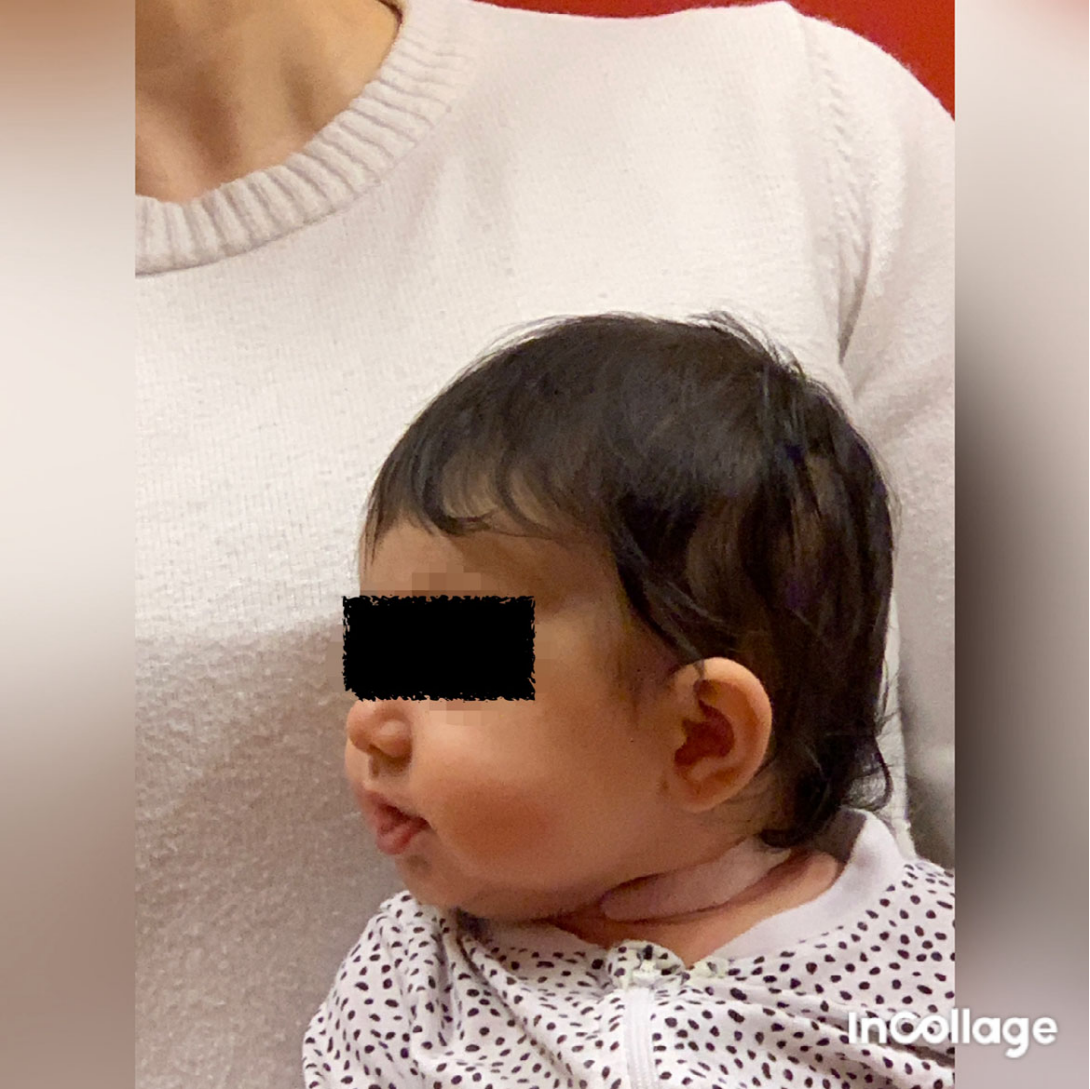
Case II rotation of the head after application of kinesiology tape, relaxing technique cross over the sternocleidomastoid muscle. I confirm that I have obtained permission to use this image from the parents of the patient included in this presentation.

Case II was admitted to Friskispraktiken, Gothenburg, Sweden was a Caucasian girl (2.5 months of age), who was prenatally in breech presentation, and was turned by the healthcare provider some weeks before delivery. At the time of delivery, she was in a cephalic presentation and was fixed in the birth canal. In August 2020 at 2.5 months of age, she visited the clinic and was referred for moderate brachycephaly. It was also obvious that the neck was affected, the sternocleidomastoid muscle was thickened bilaterally, and both active and passive rotations were affected. The head was held in flexion ( Figure 6), and active rotation was severely limited to both sides, less than 45° bilaterally ( Figure 7). Passive rotation was close to 90°, but with a clear stop, the mean normal range of rotation for infants is 110° (19). Kinesiology taping was used as a complement, relaxing technique on both sides ( Figure 8), and tape was applied across the sternocleidomastoid muscle on both sides. The parents worked with head control and stimulated motion in all the directions. They worked hard to stimulate rotation on both the left and right side. When taped, it was easier for her to move the head with a greater range of motion. When she was about five months old, the problem was solved, and she had a good head position and active rotation of approximately 80° bilaterally and passive rotation >90° bilaterally. However, after reading the case reported by Matuszewski *et al.* (2017) I decided to check her again.

At the age of nearly three years, she had an active rotation of 70° bilaterally and passive rotation of 90° bilaterally. Passive lateral flexion was full ( *i.e.*, ear to shoulder bilateral). Muscle function and strength were assessed bilaterally according to the MFS scale she scored 4 bilaterally. The marginal flexion of the head might be within the normal span. I have decided to follow her once a year for at least some years to ensure that it stays good.

## Discussion

### Case I

The combination of SMT and PT on different sides is, to my knowledge, not reported; there are probably more cases even though it might be rare. There may also be more cases than expected, but it is easy to miss if they are very mild. The current described case first met a less experienced physiotherapist, and it is understandable that she became confused. Torticollis is usually on one side, right- or left-sided. It is important to have good documentation, and if the examiner feels unsure, ask for a second opinion from a more experienced colleague. The examiner must follow the progress attentive and carefully adjust the treatment.

### Case II

As the muscle grows to about 10 cm during the child’s skeletal growth, in the first 13 years, it may be important to follow up during childhood. This will ensure that skeletal growth problems will eventually be discovered early. In my experience, adults with neglected CMT have difficulty finding physicians or physiotherapists who understand that they have experienced CMT. Even with a clear muscular string and a typical head position, some adults do not receive a correct diagnosis and treatment. For a clinician with experience in children who need surgery for CMT, an adult with the same problem is easy to recognize. However, bilateral torticollis is more confusing, and more knowledge of its long-term effects is needed.

Bilateral sternocleidomastoid tumors have been reported, with or without torticollis ( Dangi and Gwasikoti, 2020; Kumar, Prabhu, Chattopadhayay and Nagendhar, 2003; Tufano, Tom and Austin, 1999). However, not much information is available on long-term results. Neck problems are not uncommon among adults, and we should be aware that there could have been an earlier bilateral torticollis that contributed to this problem.

## Conclusions

CMT can appear in different ways and may be bilateral. To gain more knowledge about bilateral CMT, we should follow these cases over a longer period of time. It is important to communicate and discuss our experiences with each other to understand rare cases of CMT.

## Consent

Written informed consent for publication of their clinical details and clinical images was obtained from the parents of the patients.

## Data Availability

All data underlying the results are available as part of the article and no additional source data are required.
