# Bilateral congenital muscular torticollis in infants, report of two cases

**DOI:** 10.12688/f1000research.143499.2

**Published:** 2024-06-17

**Authors:** Anna Öhman

**Affiliations:** 1 Health and Rehabilitation/Physiotherapy, University of Gothenburg, Gothenburg, Sweden

**Keywords:** Torticollis, bilateral, infant, physiotherapy

## Abstract

**Background:**

Congenital muscular torticollis (CMT) is a well-known diagnosis among physiotherapists specializing in pediatric care, especially when working with infants. However, knowledge of bilateral torticollis is limited. The purpose of this article was to describe how bilateral torticollis may present itself clinically.

**Case:**

One infant with CMT with sternocleidomastoid tumor (SMT) on the right side, and some limitation in rotation towards the right side and in lateral flexion towards the left side, i.e, the muscle on the right side was shortened. While sitting with support, he tilted the head to the left and was stronger in the lateral flexors on the left side which fit well with a postural left-sided torticollis (PT). The other infant had bilateral muscular torticollis (MT), the sternocleidomastoid muscle thickened bilaterally, and both active and passive rotations were affected. The head was held in flexion, and active rotation was severely limited to both sides. For both cases the therapeutic interventions were to gain a normal range of motion (ROM) and a good posture of the head.

**Conclusions:**

CMT can appear in different ways and may be bilateral. Both infants gained good ROM and better head position, however case I still needs some training. To gain more knowledge about bilateral CMT, we should follow these cases over a longer period of time. It is important to communicate and discuss our experiences with each other to understand rare cases of CMT.

## Introduction

CMT is a common congenital musculoskeletal anomaly among infants. It results from shortening or excessive contraction of the SCM muscle. One to four weeks after birth a SMT may be visible, which consists of fibrous tissue and disappears within a few months ( Cheng, Tang and Chen, 1999; Kurtycz, Logrono, Hoerl and Heatley, 2000; Tang *et al.*, 2002; Wei, Schwartz, Weaver and Orvidas, 2001; Ohman and Beckung, 2008). The head is typically tilted towards the affected muscle, and the chin is rotated towards the other side. The true etiology of CMT have been discussed; one hypothesis is that the condition could be the sequel of an intrauterine or perinatal compartment syndrome ( Davis, Wenger, Mubarak, 1993), several studies are pointing in that direction ( Bielski *et al.*, 2006; Hardgrib, Rahbek, Möller-Madse and Maimburg, 2017; Lee *et al.*, 2011a, 2011b; Pazonyi, Kun and Czeizel, 1982; Xiong *et al*., 2019). CMT can be divided into three groups:

SMT group with a clinically palpable sternomastoid tumor
MT group with tightness of the SCM muscle but no tumor
PT group with all the clinical features of torticollis, but without the tightness or tumor of the SCM muscle ( Cheng *et al.*, 2001).

For the SMT group, intrauterine or perinatal compartment syndrome is very likely to be the cause. Mandibular asymmetry is seen during examination, Fenton *et al.* found decreased ramal height ipsilateral to the affected SCM muscle ( Fenton *et al.* 2018). It is possible that all three groups have the same cause, which is even more obvious in SMT and MT.

Infants with CMT have asymmetry in muscle function in the lateral flexors of the neck. The affected side is stronger than the other side ( Öhman and Beckung, 2005; Ohman Mardbrink, Stensby and Beckung, 2011). Assessment of side-flexor musle function in lateral head righting can be evaluated with the Muscle Function Scale (MFS) ( Ohman and Beckung, 2008; Kaplan, Coulter and Sargent, 2018). The MFS is found to be a reliable tool to test infants for CMT ( Ohman, Nilsson and Beckung, 2009; Seager; French and Meldrum, 2019). It gives information on asymmetry in head righting response. If the infant tilts the head without limited ROM and no difference in scores on the MFS, other causes than CMT must be considered. There are several differential diagnoses of CMT and some of them can be potentially life threating ( Zvi and Thompson, 2022). If an infant has an SMT on one side and tilts to the other side with higher MFS scores on that side, bilateral torticollis must be considered.

ROM in the neck can be measured with an arthrodial protractor ( Cheng *et al.*, 2001; Ohman and Beckung, 2008; Kaplan, Coulter and Sargent, 2018).

Treatment for CMT includes manual stretching, strengthening cervical muscles through positioning, handling and exercises isolating the weaker muscle, incorporating righting reactions in different positions. Developmental exercises should be incorporated to promote symmetrical movement in weight-bearing in different positions, as well as environmental adaptions and parent/caregiver education ( Kaplan, Coulter and Sargent, 2018). Kinesiology taping (KT) can be used as a complementary treatment, to give an immediate relaxing effect ( Ohman, 2012; Ohman, 2015).

Bilateral CMT is rare and not often reported, Matuszewski, Pietrzyk, Kandzierski and Wilczynsk (2017) reported a case of a child with bilateral torticollis, referred to the orthopedic department at the age of 12 years. The first symptoms appeared at preschool age. This child had severe limitations in range of motion and required bilateral surgery ( Matuszewski, Pietrzyk, Kandzierski and Wilczynsk, 2017). Babu *et al.*, presented a case report of a 19-year-old girl with congenital bilateral sternocleidomastoid contracture ( Babu, Lee, Mahadev and Lee, 2009).

A few articles describing case reports of bilateral torticollis have not been published in English ( Chiari, 1952; Kustos and Magdics, 1993; Shi *et al*., 2016; Shinoda and Yamada, 1969).

## Case I

**Figure 1.**
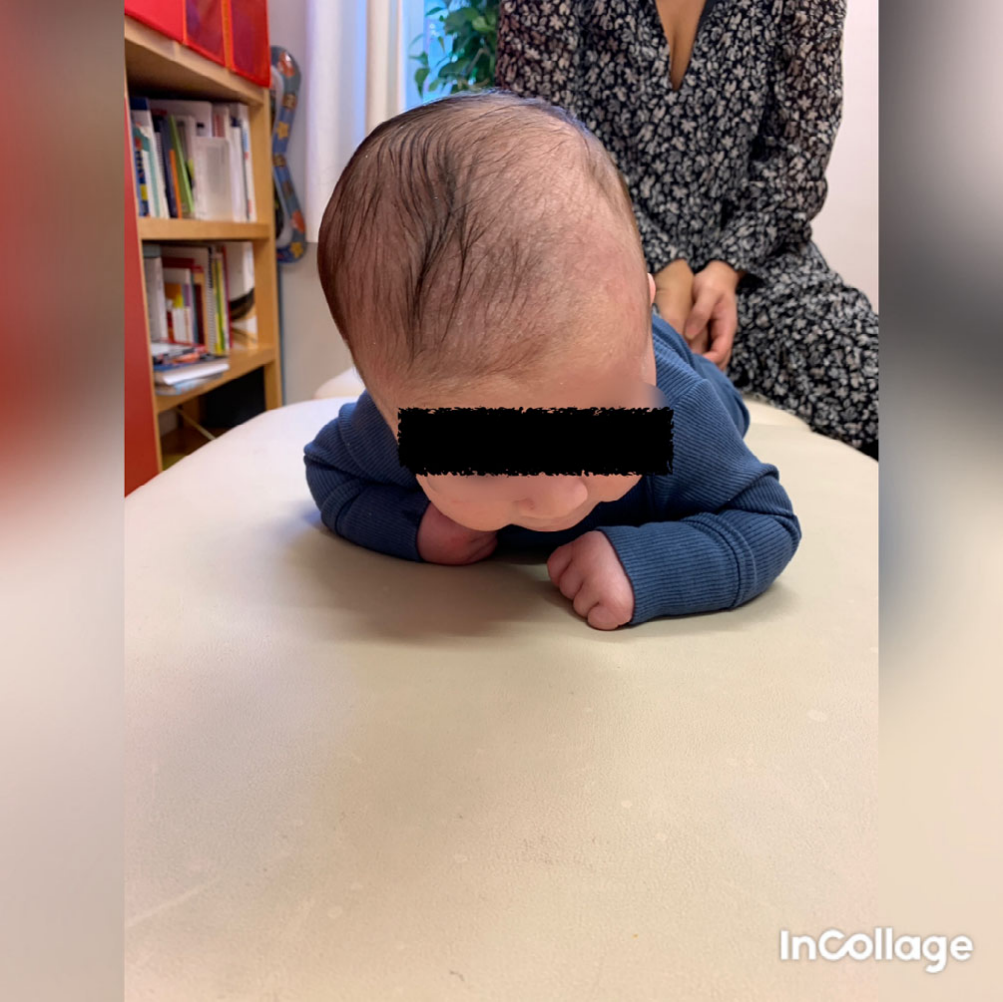
Case I, in the prone position, showed lateral flexion of the head to the right and rotation to the left. This is in agreement with right-sided torticollis. I confirm that I have obtained permission to use this image from the parents of the patient included in this presentation.

**Figure 2.**
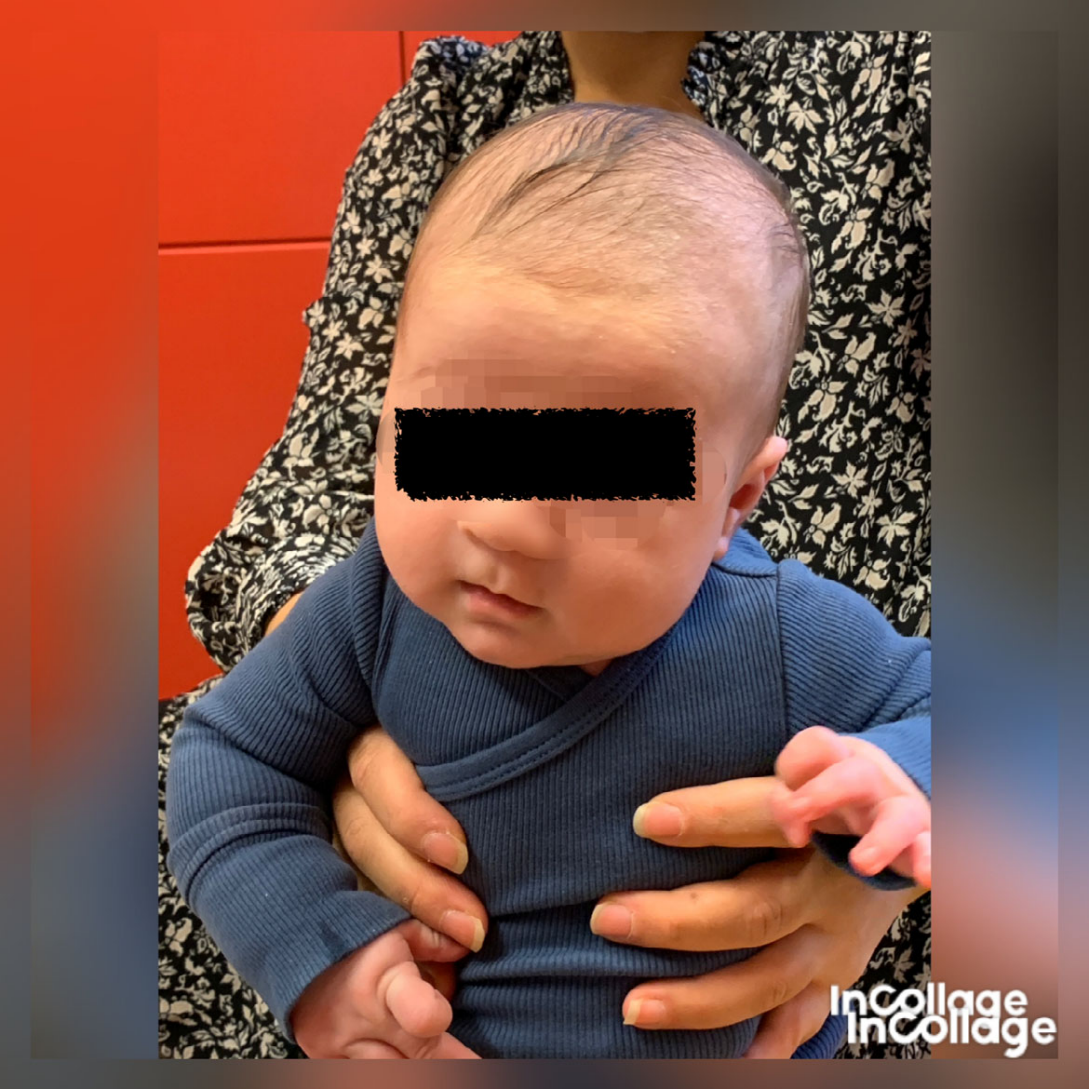
Case I in supported sitting position, lateral flexion of the head to the left and rotation to the right. This is agreeable with left-sided torticollis. I confirm that I have obtained permission to use this image from the parents of the patient included in this presentation.

**Figure 3.**
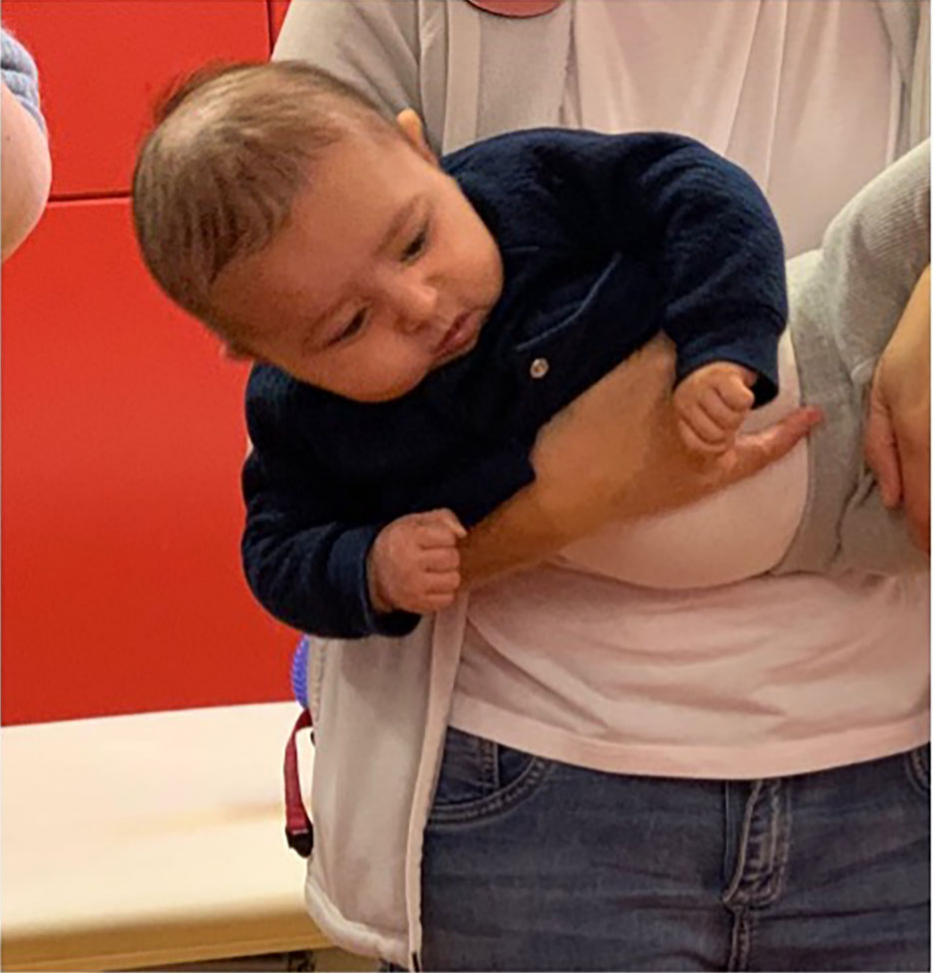
Case I test with the Muscle Function Scale (MFS) left side scoring 3, *i.e.,* left side is stronger than the right side. This is agreeable with left-sided torticollis Infants in general is righting the trunk to some extent in addition to righting the head, especially with higher scores. I confirm that I have obtained permission to use this image from the parents of the patient included in this presentation.

**Figure 4.**
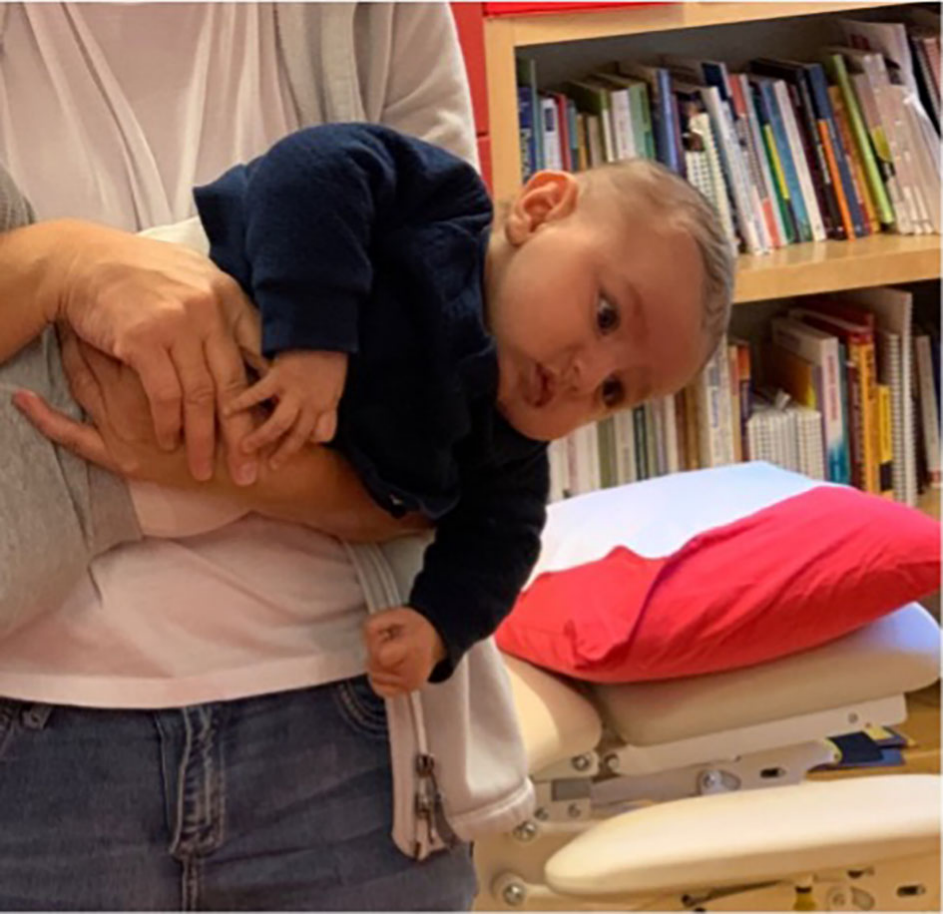
Case I test with the Muscle Function Scale (MFS) right side scoring 2, *i.e.*, right side is weaker than the left side. This is agreeable with left-sided torticollis. I confirm that I have obtained permission to use this image from the parents of the patient included in this presentation.

**Figure 5.**
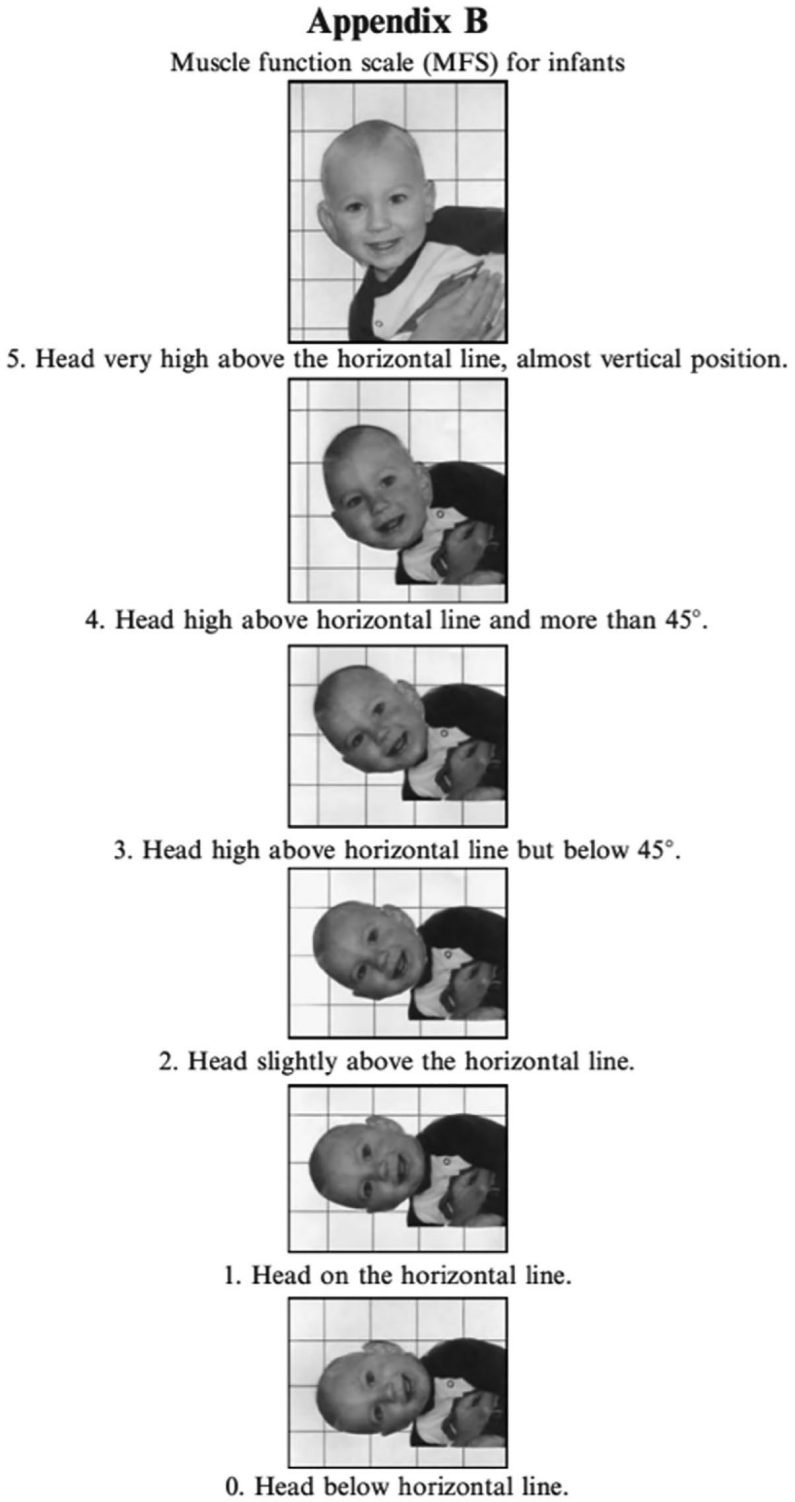
Muscle Function Scale. When the Muscle Function Scale (MFS) is used, the infant is held in a vertical position and then lowered to the horizontal position in front of a mirror. The head position is observed and both sides are tested. Scores are given according to the head position in relation to the horizontal line. The infant must be observed with the head held in the same position for five seconds to obtain the score at that level. This figure has been reproduced/adapted from Ohman *et al.* (2009). Reprinted by permission of Informa UK Limited, trading as Taylor & Francis Group, www.tandfonline.com.

Case I is a Caucasian boy who came for a second opinion since the first examiner found it confusing, when the motion, muscle function/strength, and tilting of the head did not clearly fit left- or right-sided torticollis. On clinical examination in October 2020 when he was three and a half months of age, he had bilateral torticollis. On the right side, the patient had a SMT and some limitation in rotation towards the right side and in lateral flexion towards the left side, that is, the muscle on the right side was shortened. Case I had classification grade 3 according to Kaplan et al. CMT classification grades and decision tree for 0-12 months ( Kaplan, Coulter and Sargent, 2018). When lying in the supine or prone position, he tilted the head to the right side and rotated to the left ( Figure 1), which fit well with right-sided torticollis, SMT. While sitting with support, he tilted the head to the left ( Figure 2) and was stronger in the lateral flexors on the left side ( Figures 3 and 4), which fit well with a postural left-sided torticollis (PT). ROM in the neck rotation was measured with an arthrodial protractor and muscle function was evaluated using the MFS scale ( Figure 5) ( Ohman and Beckung, 2008; Ohman, Nilsson and Beckung, 2009).

Treatment involved stretching of the SCM muscle on the right side performed by the physiotherapist three times per week. Home program with exercises; strengthening cervical muscles through positioning, handling and exrecises isolating the weaker SCM muscle. Incorporating rightning reactions in different positions. The treatment at that time gave quick results, equal strength in the lateral flexors of the neck, motion in lateral flexion, and only a marginal difference in rotation.

At the follow-up at two years of age, he had relatively good ROM and no head tilt, but he had an indication of a discreet muscular string on the right side. At the next follow-up at two and a half years of age, he had a discreet tendency to tilt the head to the right and was slightly stronger on the right side. The muscular string is still rather discreet and felt only when stretching the muscle. He has started treatment again, and the muscular string may worsen as he grows in height. The SCM muscle grows from about 4 cm in infants to 14 cm at 13 years of age, according to measures made by Jones (1968). Case I must be followed-up for a longer time, as there is a risk that he will need surgery later in life.

## Case II

**Figure 6.**
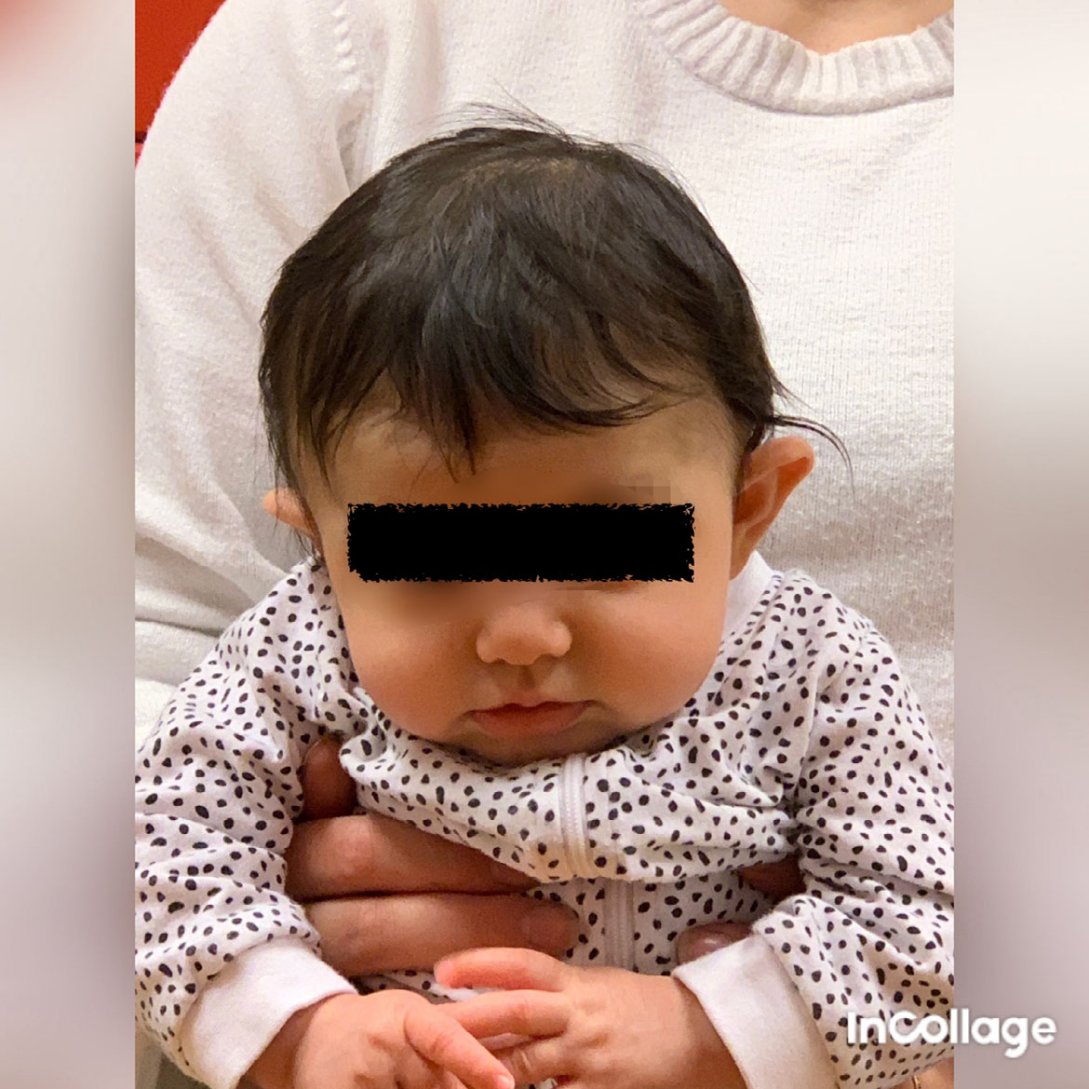
Front view of Case II, head in flexion due to bilateral torticollis. I confirm that I have obtained permission to use this image from the parents of the patient included in this presentation.

**Figure 7.**
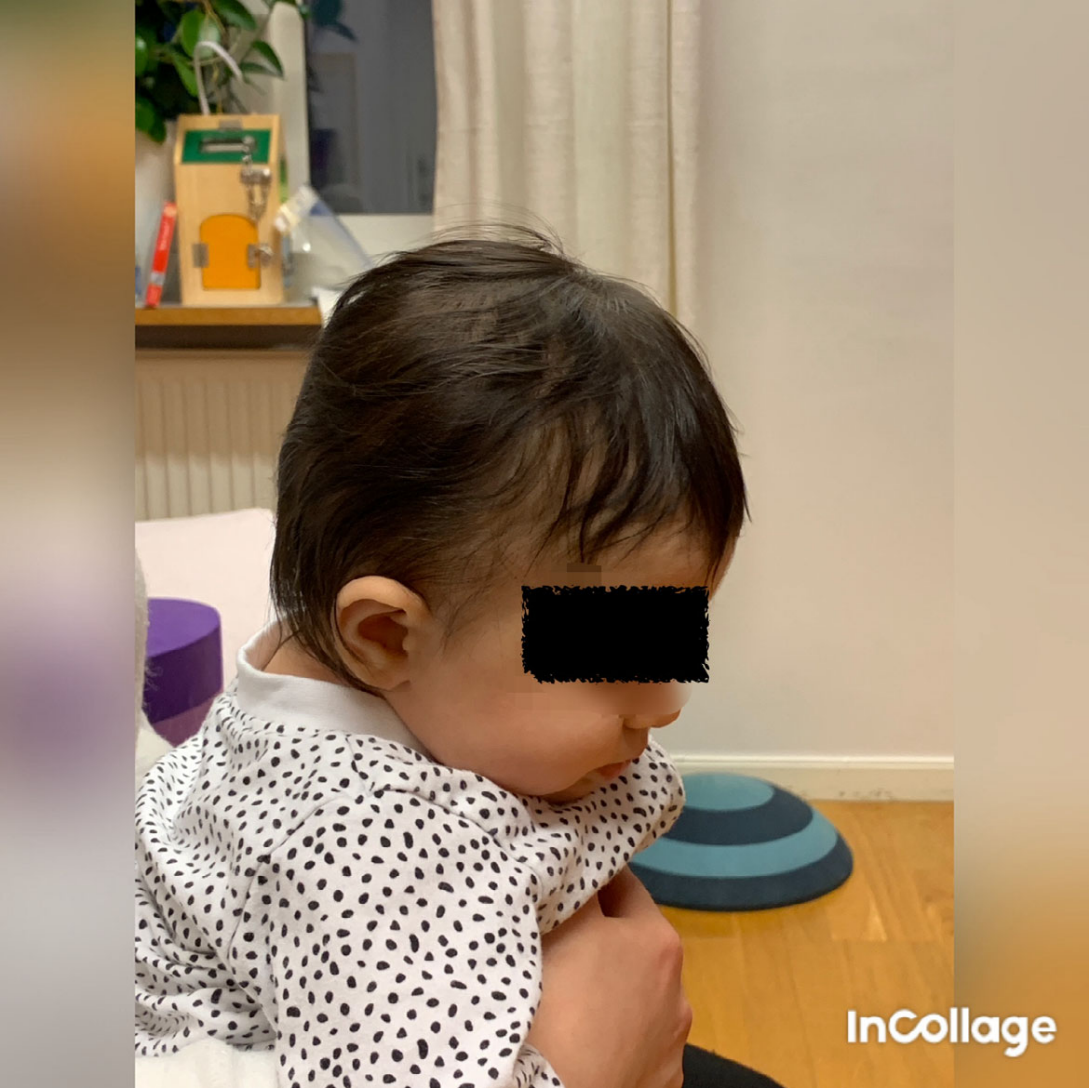
Side view of Case II, head in flexion and only attempts to rotate the head. I confirm that I have obtained permission to use this image from the parents of the patient included in this presentation.

**Figure 8.**
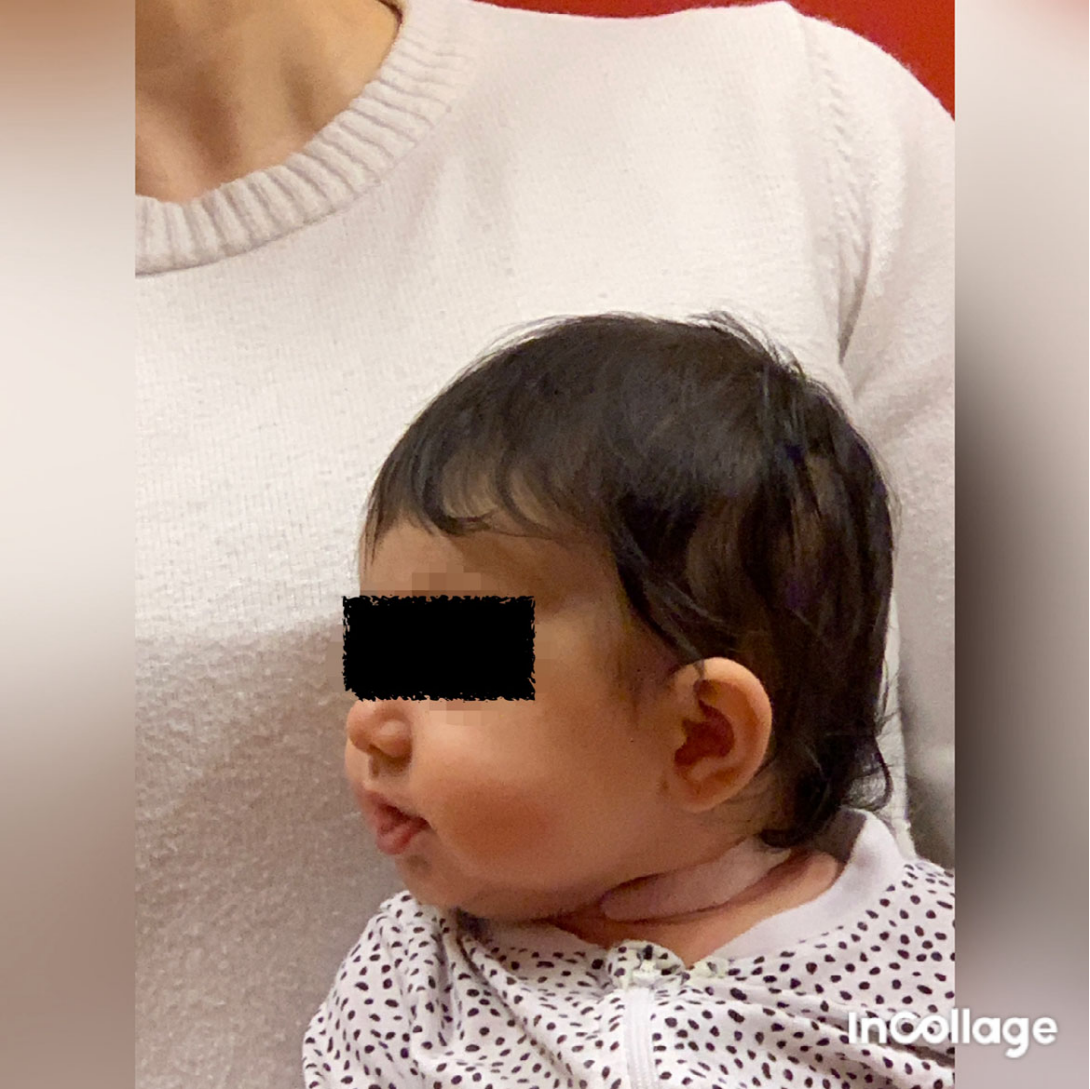
Case II rotation of the head after application of kinesiology tape, relaxing technique cross over the sternocleidomastoid muscle. I confirm that I have obtained permission to use this image from the parents of the patient included in this presentation.

Case II is a Caucasian girl who was prenatally in breech presentation, and was turned by the healthcare provider some weeks before delivery. At the time of delivery, she was in a cephalic presentation and was fixed in the birth canal. In August 2020 at two and a half months of age, she came for clinical examination, referred for moderate brachycephaly. It was also obvious that the neck was affected, the SCM muscle was thickened bilaterally, and both active and passive rotations were affected. The head was held in flexion ( Figure 6), and active rotation was severely limited to both sides, less than 45° bilaterally ( Figure 7). Passive rotation was close to 90°, but with a clear stop, the mean normal range of rotation for infants is 110° ( Ohman and Beckung, 2008). KT was used as a complement with a relaxing technique on both sides ( Figure 8), and tape was applied across the sternocleidomastoid muscle on both sides. The taping was done by the physiotherapist once a week or more often if tape came off earlier. Taping is not easy for parents without experience, infants have a short neck and a lot of skin, so taping is challenging for the inexperienced. The parents worked with the home program several times every day with head control and exercises incorporated to promote symmetrical movement in different positions. When taped, it was easier for her to move the head with a greater range of motion. When she was about five months old, the problem was solved, and she had a good head position and active rotation of approximately 80° bilaterally and passive rotation >90° bilaterally. However, after reading the case reported by Matuszewski *et al.* (2017) I decided to check her again.

At the age of nearly three years, she had an active rotation of 70° bilaterally and passive rotation of 90° bilaterally. Passive lateral flexion was full ( *i.e.*, ear to shoulder bilateral). Muscle function were assessed bilaterally according to the MFS scale she scored 4 bilaterally. The marginal flexion of the head might be within the normal span. I have decided to follow her once a year for at least some years to ensure that it stays good.

## Discussion

### Case I

The combination of SMT and PT on different sides has to my knowledge, not been reported. Even though it is rare, more cases probably exist as they are easy to miss if very mild. The combination of SMT and PT on different sides has to my knowledge, not been reported. Even though it is rare, more cases probably exist as they are easy to miss if very mild. It is important with rigorous examination and documentation to evaluate infants with torticollis, if it is bilateral CMT, the treatment must be adjusted. The MFS scale can be used in evaluating asymmetry in side-flexor muscle function in lateral head righting in PT. Healthy infants without CMT are not found to have any asymmetry on the MFS-scale ( Ohman and Beckung, 2008). For example, in clinic infants with oculat roticollis show no difference on the MFS scale (author´s experience). Still photography is a reliable method for measuring habitual head deviation from midline in infants with CMT ( Rahlin and Sarmiento, 2010). The examiner must follow progress attentively and carefully, and when needed adjust the treatment. When torticollis does not concide with right or left CMT, it must be ensured that there is no other cause. There are several differential diagnoses, some of them more serious ( Zvi and Thompson, 2022).

As the muscle grows by about 10 cm during the child’s skeletal growth, in the first 13 years, it may be important to follow up during childhood. This will ensure the early discovery of any skeletal growth problems. In my experience, adults with neglected CMT have difficulty finding physicians or physiotherapists who understand that they have experienced CMT. Even with a clear muscular string and a typical head position, some adults do not receive a correct diagnosis and treatment. For a clinician with experience in children who need surgery for CMT, it is easy to recognize an adult with the same problem. However, bilateral torticollis is more confusing, and more knowledge of its long-term effects is needed.

KT was used as complementary treatment for case II and worked well for her. With relaxing technique, KT has an immediate relaxing effect on the tense muscle ( Ohman, 2015). However, the long-term effect of KT is uncertain and needs to be further investigated ( Kaplan, Coulter and Sargent, 2018). Two studies comparing CMT treatment with and without KT came to opposite conclusions about the effect of KT ( Giray *et al.*, 2017; Hussein, Ali and El-Meniawy, 2019).

Bilateral sternocleidomastoid tumors have been reported, with or without torticollis ( Dangi and Gwasikoti, 2020; Kumar, Prabhu, Chattopadhayay and Nagendhar, 2003; Tufano, Tom and Austin, 1999). However, not much information is available on long-term results. Neck problems are not uncommon among adults, and we should be aware that there could have been an earlier bilateral torticollis that contributed to this problem.

## Conclusions

As bilateral CMT is rare and might be confusing, it needs to be further evaluated. Critical examination and documentation when evaluating CMT is important to give the right treatment when bilateral and also to make sure that a differential diagnosis is not missed by mistake. The need for longer follow-up for infants with bilateral CMT should be considered, to learn if there are any long-term problems.

## Consent

Written informed consent for publication of their clinical details and clinical images was obtained from the parents of the patients.

## Data Availability

All data underlying the results are available as part of the article and no additional source data are required.
