# Supplementary material for: Bilateral congenital muscular torticollis in infants, report of two cases
Source: F1000Res. 2025 May 19;13:211. Originally published 2024 Mar 21. [Version 3] doi: 10.12688/f1000research.143499.3 (PMC12120420; doi:10.12688/f1000research.143499.3)
Supplement: Peer review history

# Reviewer report: Reviewer response for version 3

**DOI:** 10.5256/f1000research.182056.r385845

**Published:** 2025-05-28

**Authors:** Nidhi Sharma, Parveen Kumar

**Affiliations:** 1 Maharishi Markandeshwar (Deemed to be University), Ambala, India; 2 Pal Healthcare, Haryana, India

Dear Authors,

 You have done a commendable work. All the revisions have been made and no further comments are needed for the correction or modification in the present manuscript.

 Thanks

Are enough details provided of any physical examination and diagnostic tests, treatment given and outcomes?

Partly

Is the background of the cases’ history and progression described in sufficient detail?

Yes

Is sufficient discussion included of the importance of the findings and their relevance to future understanding of disease processes, diagnosis or treatment?

Yes

Is the conclusion balanced and justified on the basis of the findings?

Yes

Reviewer Expertise:

Neuro-coordination, physiotherapy, Neurological rehabilitation, Musculoskeletal rehabilitation, Outcome measures, Quantitative and qualitative research

We confirm that we have read this submission and believe that we have an appropriate level of expertise to confirm that it is of an acceptable scientific standard.

# Reviewer report: Reviewer response for version 2

**DOI:** 10.5256/f1000research.166994.r376878

**Published:** 2025-05-09

**Authors:** Nidhi Sharma, Parveen Kumar

**Affiliations:** 1 Maharishi Markandeshwar (Deemed to be University), Ambala, India; 2 Pal Healthcare, Haryana, India

Summary of the Article

 The author presents two case reports of infants with bilateral congenital muscular torticollis (CMT), a rare condition that can complicate diagnosis and management compared to the more common unilateral form. The manuscript aims to highlight the clinical presentation, assessment using the Muscle Function Scale (MFS), management including physiotherapy and kinesiology taping (KT), and short-term follow-up outcomes. The article also discusses differential diagnosis and emphasizes the need for long-term monitoring.

 The author has provided clear descriptions of the cases, useful clinical photographs, and a practical discussion of challenges faced during diagnosis and treatment. The subject is relevant and contributes important observations to the relatively sparse literature on bilateral CMT.

 ROM measurements are incompletely reported; secondary musculoskeletal features are not described; home program details need clarification.

 Major Points to Address

- Provide precise ROM values for active and passive movements.
- Clarify the ROM measurement technique.
- Explicitly report on secondary musculoskeletal features (e.g., head shape, trunk asymmetry).
- Detail the home exercise program, particularly the stretching frequency.
- Acknowledge or correct inconsistencies in MFS photograph interpretation.

Are enough details provided of any physical examination and diagnostic tests, treatment given and outcomes?

Partly

Is the background of the cases’ history and progression described in sufficient detail?

Yes

Is sufficient discussion included of the importance of the findings and their relevance to future understanding of disease processes, diagnosis or treatment?

Yes

Is the conclusion balanced and justified on the basis of the findings?

Yes

Reviewer Expertise:

Neuro-coordination, physiotherapy, Neurological rehabilitation, Musculoskeletal rehabilitation, Outcome measures, Quantitative and qualitative research

We confirm that we have read this submission and believe that we have an appropriate level of expertise to confirm that it is of an acceptable scientific standard, however we have significant reservations, as outlined above.

# Reviewer report: Reviewer response for version 2

**DOI:** 10.5256/f1000research.166994.r301321

**Published:** 2024-07-23

**Authors:** Leo van Vlimmeren

**Affiliations:** 1 Radboud University Medical Center, Nijmegen, The Netherlands

This manuscript describes 2 cases with a rare, bilateral congenital muscular torticollis.

 The focus seems to be mainly on the primary abnormalities resulting from the affected SCM on both sides: the passive, angular, cervical ROM (less on the active ROM) and on the muscle strength of the cervical lateral flexion (not on the rotation). This may be too limited in a description of the clinical manifestations.

 Case 1 is recognizable. Also regarding the quick recovery. In the treatment, stretching of the SCM is mentioned by the physiotherapist 3 times a week. In my opinion, it is customary to have this carried out by the parents several times a day. Is this also done or was it a conscious choice to only stretch 3 times a week (which may not be effective, I think). The ROM is measured with the Arthrodial Protractor, but is not further explained in the description. The descriptions therefore remain vague and lack some conviction.

 Case 2 is atypical for CMT. Such a rapid recovery from CMT (from age 2.5 to 5 months) is unlikely and seems to indicate a different etiology. Has cervical spine imaging been done? Could there have been cervical pain, for example caused by birth? Was there a (double-sided) SMT or was the SCM completely thickened or was there strand (string) formation?

 As reviewer 2 already mentioned, the starting position of the child in Figure 3 is not correct. The trunk is not kept horizontal. This is not the case with all photos in Figure 5. This should be adjusted as this often leads to ambiguity in the implementation and interpretation of the MFS.

 I miss the description of all secondary abnormalities, as are common with CMT:

 • Shape of the head, face, jaws.

 • Position of the ears, orbits, teeth.

 • Position of the head at rest (lying or vertical position).

 • Head deviation sideways.

 • Shoulderstand: one- or two-sided elevation. Shoulder retraction.

 • Spinal alignment. Scoliosis?

 • Motor development. Asymmetry of trunk, arm and leg movements.

 All these abnormalities can occur at a very young age and partly determine the choices of diagnosis and treatment.

 Case 1 discusses a possible indication for surgery. On what basis would a decision be made to undergo surgery? Only primary or also secondary abnormalities due to CMT? And what should the operation consist? Mono or bipolar release? And what follow-up treatment would be necessary?

Are enough details provided of any physical examination and diagnostic tests, treatment given and outcomes?

Yes

Is the background of the cases’ history and progression described in sufficient detail?

Yes

Is sufficient discussion included of the importance of the findings and their relevance to future understanding of disease processes, diagnosis or treatment?

Yes

Is the conclusion balanced and justified on the basis of the findings?

Yes

Reviewer Expertise:

Pediatric physical therapy especially torticollis, skull deformation.

I confirm that I have read this submission and believe that I have an appropriate level of expertise to confirm that it is of an acceptable scientific standard, however I have significant reservations, as outlined above.

# Reviewer report: Reviewer response for version 1

**DOI:** 10.5256/f1000research.157168.r264882

**Published:** 2024-05-09

**Authors:** Lucie Pelland

**Affiliations:** 1 Idaho State University, Pocatello, Idaho, USA

The topic is relevant to practice as bilateral torticollis is a rare clinical presentation. As stated by the author, a bilateral presentation can lead to uncertainty in diagnosis. 

 The Introduction could be improved in terms of the logical flow of information provided.  As an example, possible causes of CMT are presented, including SCM tumor and intra-uterine or perinatal compartment syndrome. I suggest that these possible causes be related to the three clinical CMT groups presented. This would tie in how compartment syndrome relates to this classification. The author should also consider introducing the MFS in the introduction and present a rationale on how the MFS, within a standardized clinical assessment, could provide an approach to avoid a missed diagnosis of bilateral CMT. The introduction should end with the statement of aim provided in the abstract - which could be modified as follows: Our purpose was to describe the clinical presentation of bilateral CMT to improve diagnosis. 

 In the cases, there is no need to include the clinic where the clinical evaluation was performed. This information is provided in the author's contact. The language used should be formalized for scientific writing (e.g., 'I met this boy in October 2020' should be stated as 'On clinical examination in October 2020'). 

 It might be clearer to align the figures for the two cases with the MFS and to provide the score for each Figure. For Case I, I would place Figures 3 and 4 side by side to more clearly show that the left SCM is stronger than the right. As well, in Figure 1, it is not clear if the head position may have been influenced by the asymmetric positioning of the upper limbs in prone support. 

 In case II, the HEP for parents is not clear - as well, was kinesiotaping applied at home? This would be important information for clinicians treating infants with bilateral CMT. For Case II, the figures do clearly show the forward head flexion. For clarity, I would suggest, again, that all positions in the MFS should be shown. This would help to problem solve the clinical presentation (which is the aim of the paper). 

 The quality of the images in Figure 5 needs to be improved.   

 The discussion repeats information that has been presented in the cases and in the introduction. I suggest that the discussion should focus on providing the structured approach, including the MFS, that should be used to avoid 'missing the diagnosis' of bilateral CMT. Treatment could be summarized in alignment with the clinical findings, providing a translation of findings to practice to improve patient outcomes. 

 General Comments:

 1. There is a need for improving grammar and sentence structure throughout.

 2. In the abstract, avoid using abbreviations that are only used once. 

 3. Specificity of terminology needs to be verified (e.g., in the abstract, 'how bilateral torticollis can appear in infants' would be better stated as 'how bilateral torticollis may present clinically'. 

 4. Once a term is abbreviated (e.g., SCM for sternocleidomastoid) it should then be used consistently. 

 Overall, this is a clinically relevant paper which focuses on an area of pediatric practice which requires further evidence.

Are enough details provided of any physical examination and diagnostic tests, treatment given and outcomes?

No

Is the background of the cases’ history and progression described in sufficient detail?

Partly

Is sufficient discussion included of the importance of the findings and their relevance to future understanding of disease processes, diagnosis or treatment?

No

Is the conclusion balanced and justified on the basis of the findings?

No

Reviewer Expertise:

Biomechanics, neuroscience, pediatrics

I confirm that I have read this submission and believe that I have an appropriate level of expertise to state that I do not consider it to be of an acceptable scientific standard, for reasons outlined above.

# Response

**Published:** 2024-05-23

**Authors:** Anna Öhman

**Affiliations:** Health and Reabilitation, University of Gothenburg, Gothenburg, Sweden

Thanks for all valuable comments

 made changes and added information, hopefully made the manuscript better.

 New grammar check was also done

# Reviewer report: Reviewer response for version 1

**DOI:** 10.5256/f1000research.157168.r264886

**Published:** 2024-05-02

**Authors:** Kimberly B. Castle

**Affiliations:** 1 University of North Georgia, Dahlonega, Georgia, USA

This is a topic of interest to many pediatric physiotherapists and pediatricians. There is benefit in disseminating the information from these cases.

 Details of the second case are much more specific than those of the first case. It is difficult to understand the exact extent of the first infant's passive limitation even with the photographs as there are no measures or estimates of limitations beyond the Muscle Function Scale.  

 As Dr. Ohman is the creator of the MFS, the evaluations using the tool are deemed to be accurate. However, the photos appear to show a difference in the trunk alignment of the infant when held to each side. Is the reader to assume that the infant is righting the trunk to some extent in addition to righting the head to the left (first MFS photo)?  If not, is there a photo in which the therapist's hands support the trunk similarly that would allow comparison? Perhaps a brief explanation of the difference would be helpful for interpretation.

 In the second case, was the flexed neck posture true neck flexion or rather a forward head posture with cervical flexion & capital extension? It is difficult to assess from the lateral photo and would be interesting to know if SCM were the only muscles that were tight or if there was increased tension in posterior neck musculature as well.

 Finally, the discussion including typical growth in the muscular and skeletal growth throughout childhood was helpful. It would also be helpful to include a synopsis of how passive and active components impacted these children's posture and active movement depending on posture, position and the effects of gravity.

Are enough details provided of any physical examination and diagnostic tests, treatment given and outcomes?

Partly

Is the background of the cases’ history and progression described in sufficient detail?

Partly

Is sufficient discussion included of the importance of the findings and their relevance to future understanding of disease processes, diagnosis or treatment?

Partly

Is the conclusion balanced and justified on the basis of the findings?

Yes

Reviewer Expertise:

Pediatric physiotherapy with specific emphasis on core trunk and neck control including torticollis

I confirm that I have read this submission and believe that I have an appropriate level of expertise to confirm that it is of an acceptable scientific standard, however I have significant reservations, as outlined above.

# Response

**Published:** 2024-05-23

**Authors:** Anna Öhman

**Affiliations:** Health and Reabilitation, University of Gothenburg, Gothenburg, Sweden

Author response:

 Thanks for all valuable comments, there are a lot more to discuss about CMT,

 Your review add some new thoughts.
